# Neutrophils: tissue and circulating signatures of pediatric non-alcoholic fatty liver disease

**DOI:** 10.3389/fcell.2023.1336033

**Published:** 2024-01-04

**Authors:** Maria Oana Săsăran, Carmen Muntean, Ancuța Lupu, Vasile Valeriu Lupu

**Affiliations:** ^1^ Department of Pediatrics 3, George Emil Palade University of Medicine, Pharmacy, Science, and Technology of Targu Mures, Targu Mures, Romania; ^2^ Department of Pediatrics 1, George Emil Palade University of Medicine, Pharmacy, Science, and Technology of Targu Mures, Targu Mures, Romania; ^3^ Department of Pediatrics, University of Medicine and Pharmacy Gr. T. Popa Iași, Iași, Romania

**Keywords:** neutrophil, children, NAFLD, NLR, inflammation

## Abstract

The recent rise in non-alcoholic fatty liver disease (NAFLD) among children and adolescents led to a thorough investigation of the peculiarities of the cellular infiltrate which characterize the disease at young ages. This review aims to highlight the key involvement of neutrophils in the pathogenesis of pediatric NAFLD and the potential biomarker role of neutrophil-to-lymphocyte ratio (NLR) in the same pediatric disorder. Neutrophils, which are first responders to inflammation, constitute an abundant component of an infiltrate which is particularly disposed within the portal area of children with NAFLD. The involvement of neutrophils in triggering liver fibrosis has been related amongst others to reactive oxygen species (ROS) production, to the stimulation of hepatic stellate cells, and to their synthesis of neutrophil elastase. As immune imbalance characterizes NAFLD, potentially emerging non-invasive biomarkers such as NLR have been proposed for the detection and prognosis of NAFLD. In adults, several studies asserted the role of NLR in the prediction of advancing liver fibrosis and mortality in subjects with NAFLD. In children, data is scarce with contradicting findings, as some studies failed to identify significant shifting in NLR values in children with NAFLD when compared with obese controls without liver impairment. However, NLR seems to significantly increase in children with obesity and different degrees of NAFLD when compared to healthy counterparts and their changes seem to be reversible with weight loss. Still, paucity of pediatric studies calls for future research addressing the role of NLR in predicting NAFLD development and progression in children with obesity.

## 1 Introduction

Non-alcoholic fatty liver disease (NAFLD), the most common form of liver disease, is characterized by the accumulation of triglycerides inside hepatocytes, in the absence of alcohol consumption history and of other etiological factors responsible for liver injury (Matteoni et al., 1999; Angulo, 2007). The interrelation between NAFLD and metabolic dysfunctions suggests the involvement of intricating pathophysiological mechanisms in the genesis of this disease, which can lead to hepatic complications and adverse cardiovascular outcomes (Mantovani et al., 2021). In terms of liver impairment, NAFLD can encompass a large spectrum, ranging from steatosis to advanced fibrosis and liver cirrhosis (Angulo, 2002). The quest for the identification of non-invasive biomarkers that can prematurely detect NAFLD, as well as its complications, has recently focused on inflammatory markers (Tarantino et al., 2009). However, study outcomes have been surrounded by controversy, as negative and null correlations between these potential markers and NAFLD have also been proven (Viglino et al., 2017; Cabré et al., 2019).

NAFLD represents a major health burden in children, being associated with a more increased risk of long-term complications. The Western dietary pattern represents a major risk factor of NAFLD, whose incidence ranges between 8% and 16% in adolescents (Raj et al., 2023). Children with NAFLD have no symptoms in the early stages, and the clinical picture of pediatric NAFLD unveils when significant liver damage had already developed. Pediatric patients with NAFLD typically report nonspecific symptoms such as lethargy, malaise, or diffuse abdomen discomfort, particularly in the upper right quadrant, which may be associated with progressive fibrosis and hepatomegaly (in up to 50% of cases). Acanthosis nigricans is a common sign of hyperinsulinemia, which has been found in up to half of children with biopsy-proven NAFLD (Mărginean et al., 2021).

A nationwide study conducted in Sweden proved that children and young adults with NAFLD which has been histologically confirmed present overall higher mortality rates, and are more prone towards developing cancers, liver and cardiometabolic diseases (Simon et al., 2021). Within another study, longitudinal follow-up of 20 years in a pediatric cohort diagnosed with NAFLD showed that a significant number of patients progress towards end-stage liver disease and require liver transplantation (Feldstein et al., 2009). Although the current gold standard diagnostic method in pediatric NAFLD is represented by liver biopsy, several non-invasive biomarkers are currently being proposed for its early recognition (Vos et al., 2017; Jayasekera and Hartmann, 2023). However, their utility is questionable, as it is presumed that only their combined use with other laboratory tests or imaging investigations can accurately quantify degree of liver fibrosis and can monitor disease progression (Mosca et al., 2020).

Obesity fosters a systemic low grade inflammation which can lead to a baseline increase in leukocyte numbers in children when compared to healthy counterparts, even in the absence of an infectious process (Shi et al., 2017). Obesity-related complications, including dyslipidemia, metabolic syndrome and insulin resistance have been associated with increase in peripheral white blood cell counts in young adults, which had been previously proven to reflect systemic inflammation (Hotamisligil, 2006; Yoshimura et al., 2015). Metabolic syndrome and insulin resistance seemed to mediate the increase in WBC counts in NAFLD patients as well, according to Lee et al. (2010). Some other authors argue this theory, suggesting that although insulin resistance plays an important role in the pathogenesis of NAFLD, systemic inflammation links WBC increase to NAFLD (Zhang et al., 2019). An independent association between WBC count and NAFLD has also been demonstrated, which emerged as an easily accessible marker of systemic inflammation (Lee et al., 2010; Yu et al., 2018). With NAFLD, the excessive accumulation of free fatty acids inside the hepatocytes promotes production of cytokines such as interleukin (IL)-1, IL-6 and tumor necrosis factor-α (TNF-α), which will in turn enhance leukocyte recruitment (Wieckowska et al., 2008). The WBC counts increase proportionally with the severity of hepatic steatosis, according to Chao et al. 2022). Increase in WBC numbers has also been related to incidental NAFLD among Chinese studies (Chung et al., 2016; Wang et al., 2016). Hence, the next logical step was to address how the variation of different WBC subtypes relates to NAFLD, especially given the neutrophil and lymphocyte variation which is particularly susceptible to systemic inflammation and to cytokines secreted by the adipocyte (Zhang et al., 2019). Some other WBC subtypes, such as peripheral monocytes, were also reported to be the only WBC subtypes which significantly increase in NAFLD, within another study (Kim et al., 2011).

Neutrophils are first responder cells to inflammation and they interact with antigen-presenting cells and promote macrophage recruitment as well, which are responsible for the induction and maintenance of a chronic inflammatory status within various tissues, such as the liver (Nathan, 2006; Mantovani et al., 2011). In mice, the depletion of neutrophils with monoclonal antibodies which target a glycosylphosphatidylinositol (GPI)–anchored protein on the neutrophil surface, weight gain, accumulation of triglycerides within the hepatocytes, the activity of proteins involved in hepatic pro-inflammatory and pro-fibrotic pathways and regulated glycemia (Ou et al., 2017). Hence, the involvement of neutrophils in the development of metabolic syndrome, hepatic inflammation and fibrosis has been well documented. This review aims to highlight the key involvement of neutrophils in the pathogenesis of pediatric NAFLD and the potential biomarker role of the commonly used WBC subtype-derived parameter of inflammation, neutrophil-to-lymphocyte ratio (NLR), within the same hepatic condition in children.

## 2 Neutrophils and their involvement in non-alcoholic fatty liver disease pathogenesis

**FIGURE 1:**
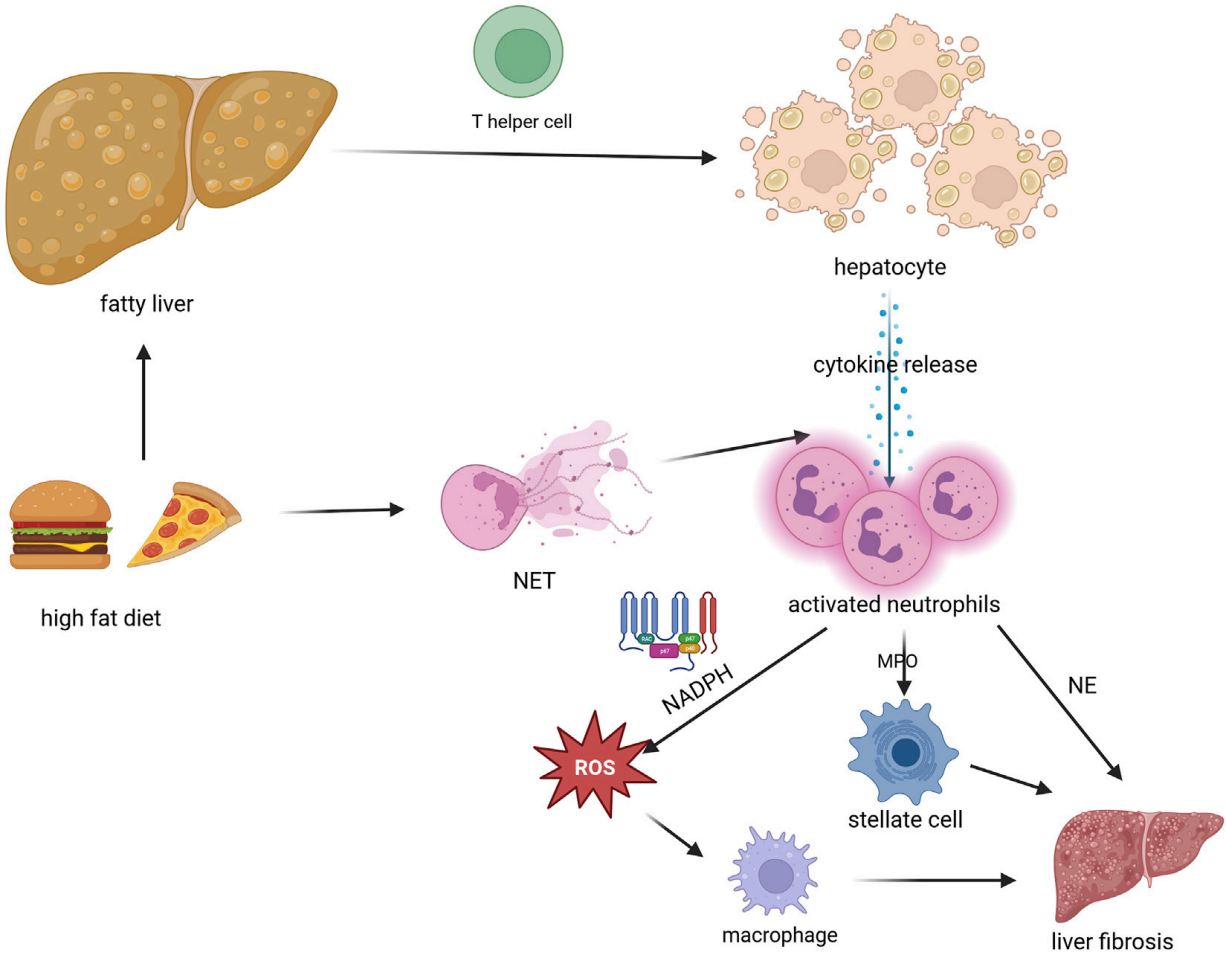
The involvement of neutrophils in the development of NAFLD-associated liver fibrosis Created with BioRender.com (https://biorender.com/). Legend: MPO- myeloperoxidase; NADPH- nicotinamide adenine dinucleotide phosphate oxidase; NE-neutrophil elastase; NET-neutrophil extracellular trap; ROS- reactive oxygen species. Accumulation of triglycerides inside hepatic cells will make them susceptible to the action of T helper cells, thus stimulating cytokine release, which in term will promote neutrophil infiltration and activation. Intake of fatty foods will stimulate the production of NETs, which also enhance cytokine synthesis. Activated neutrophils will promote the activation of NADPH and consequently ROS synthesis, which will stimulate macrophage function. The MPO secreted by the neutrophils activates hepatic stellate cells. Macrophages, hepatic stellate cells and NE are all involved in the development of liver fibrosis.

The pathogenesis of NAFLD was firstly attributed to the “two hit hypothesis” in both children and adults (Dowman et al., 2010). Initially, an accumulation of triglycerides among the hepatocytes and insulin resistance both represent the “first hit”. The gradual liver fat accumulation in over 5% of the liver surface will eventually lead to hepatic steatosis, if no lifestyle change intervenes (Wang et al., 2008). The fatty liver will be an easy target for the “second hit,” which is characterized by the release of mediators of inflammation such as cytokines and adipokines and the induction of oxidative stress (Ratziu et al., 2010; Berardis and Sokal, 2014). T helper cells seem to particularly play an important role in the induction of the tissue inflammatory process, through the cytokines they secrete, such as interleukin (IL)-17, IL-21, IL-22, and TNF*α*, which will afterwards induce the secretion of other proinflammatory mediators and recruitment of neutrophils, to the site of inflammation (Ouyang et al., 2008). The exact functional role of the neutrophils in the development of NASH and NAFLD still needs to be investigated, although evidence suggests that this type of cells are actively involved in the pathophysiological chain of events and are effectors of innate immunity (Hübscher, 2006; Macek Jilkova et al., 2016). The so far proven involvement of neutrophils in the development of NAFLD-associated liver fibrosis has been highlighted through Figure 1. In an animal model, intake of free fatty acids stimulated the production of neutrophil extracellular traps (NETs), which enhanced inflammatory cell infiltration and synthesis of pro-inflammatory cytokines (van der Windt et al., 2018). The activation of neutrophils has been hypothesized to be involved in the production of reactive oxygen species (ROS), through the activation of the nicotinamide adenine dinucleotide phosphate oxidase (NADPH) (Hwang et al., 2021). Neutrophil secretion of myeloperoxidase (MPO) is directly involved in the hepatocyte apoptosis and in triggering hepatic stellate cell function, which will promote liver fibrosis (Rensen et al., 2009; Pulli et al., 2015). ROS are also responsible for the recruitment and activation of macrophages, thus accentuating hepatocyte injury and enhancing the release of inflammatory cytokines (Hwang et al., 2021). The involvement of neutrophils in the pathogenesis of NAFLD is further ascertained by the results of an experimental treatment with anti-neutrophil antibodies, carried out in obese mice, which produced an improvement in metabolic-associated liver dysfunction (Ou et al., 2017). Neutrophils also seem to play a role in the resolution of liver inflammation, through enhancement of microRNA-223 expression, as proven on an animal model (Calvente et al., 2019).

The release of the neutrophil elastase (NE) during inflammation has also been regarded as a key effector in the pathogenesis of NAFLD (Mirea et al., 2019). NE binds to alpha-1-antitrypsin (AAT) in the extracellular space, which will in turn inhibit NE. The NE to AAT ratio is higher in NAFLD patients than in healthy controls and is associated to the severity of NASH, according to Zang et al. (2016). Furthermore, within another study, higher serum levels of NE were indicative of advanced NAFLD and liver fibrosis (Mirea et al., 2019). The experimental deletion of the Elane gene, encoding NE, increased hepatic sensitivity to insulin and reduced expression of the liver tissue-related inflammatory genes, in mice fed with a diet with high fat content (Talukdar et al., 2012). NE also plays a role in NET formation, which supports the inflammatory process and the harmful effects of the neutrophils upon liver metabolism (Papayannopoulos, 2018).

## 3 Characteristics of neutrophil infiltration in pediatric non-alcoholic fatty liver disease

The presence of neutrophils as part of lobular inflammation was more frequent in cases of alcoholic steatohepatitis than in non-alcoholic steatohepatitis (NASH), according to a study conducted in India. Moreover, the authors described the lack of extensive neutrophil infiltration within the portal tracts as characteristic of NASH (Singh et al., 2010). However, in children, a particular abundance of inflammatory infiltrate largely composed of neutrophils, alongside with histiocytes, lymphocytes and Kupffer cells within the portal area has been described, with the zone 1 NASH pattern being commonly found (Carter-Kent et al., 2009; Trandafir et al., 2020). As a matter of fact, Schwimmer et al. described two distinct phenotypes of pediatric NAFLD: the “adult” type, in which steatosis prevales in zone 3 hepatocytes, surrounding the central veins, and is accompanied by lobular inflammation, perisinusoidal fibrosis and ballooning. and the “pediatric” type in which ballooning is absent, whereas portal inflammation and fibrosis are found (Schwimmer et al., 2005). In pediatric NAFLD, Nobili et al., 2006 described an immune cell infiltration which was largely composed of lymphocytes and neutrophils, and scattered granulomas in which eosinophils and mononuclear histiocytic cells accompany the aforementioned cells. The number of infiltrating neutrophils was found to be positively associated with ROS production by peripheral polymorphonuclear cells (Ferreyra Solari et al., 2012). Other peculiarities of the cellular infiltrate have been described in children with NASH as well, distinctive of adult NASH. CD8^+^ T cells alongside interferon-gamma (IFN-γ) dominate the hepatic microenvironment of pediatric NASH over cellular subpopulations such as CD4^+^ and CD20^+^ T cells. In adults, CD8^+^ cells only make up for a minor part of Natural Killer (NK) cells (Ferreyra Solari et al., 2012).

## 4 Neutrophil-to-lymphocyte ratio: a predictor of prognosis and mortality in non-alcoholic fatty liver disease

At the cellular level, immune imbalance seems to characterize NAFLD, with NLR above a certain cutoff value being associated with poor prognosis of the disease (Alkhouri et al., 2012; Paquissi, 2016). NLR mirrors chronic, systemic inflammatory response and has been related to malignancies, cardiovascular disease and liver diseases such as NAFLD, viral hepatitis, liver cirrhosis and hepatocellular carcinoma (Ommen et al., 1998; Avanzas et al., 2004; Walsh et al., 2005; Alkhouri et al., 2012; Biyik et al., 2013; Motomura et al., 2013; Liu et al., 2014; Shavakhi et al., 2022). Neutrophil increase reflect ongoing, persistent inflammation, in the context of their migration from vessels towards peripheral tissues, triggered by chemotactic agents and adhesion molecules (Nieman et al., 1999). Lymphocytes are involved in the regulation of the hypothalamic-pituitary-adrenal axis, their decrease being associated with an enhancement of cortisol production and physiological stress among advanced heart failure patients (Ommen et al., 1998). Moreover, lymphocytes are abundant among the adipocytes and control the macrophage release of inflammatory mediators (Donath and Shoelson, 2011).

Besides inflammation, another hypothesis sustains the role of a hormonal mechanism that is responsible for the increase in NLR levels with NASH development (Yilmaz et al., 2015). Induction of the 11β-Hydroxysteroid dehydrogenase type 1 (11β-HSD1) enzyme occurs, with advancement of inflammatory process and hepatocyte injury, as NAFLD progresses (Ahmed et al., 2012). 11β-HSD1 mediates the transformation of inactive cortisone into active cortisol, and the resulting relative hypercortisolemia will lead to leukocytosis, neutrophilia and lymphopenia. Thus, NLR will increase as well (Yilmaz et al., 2015).

A gradual increase in NLR has been noted in relation to advancing hepatosteatosis in patients with type 2 diabetes, as quantified through liver ultrasonography (Kahraman et al., 2016). Research has showed that NLR correlates with histological severity of NAFLD as well, and can help in identifying patients with advanced disease. In a cohort of adult patients with NAFLD, NLR was significantly associated with advanced inflammation and fibrosis (Khoury et al., 2019). NLR is a simple, non-invasive tool, which can distinguish NASH from simple steatosis and healthy subjects (Rafat et al., 2015). Although its specificity in detecting NASH and severe fibrotic liver tissue is not optimal, NLR can apparently be used in combination with other markers/imagistic tools to identify advancing NAFLD (Alkhouri et al., 2012). Moreover, NLR emerged as a more efficient biomarker in the prediction of NASH and liver fibrosis than C reactive protein (CRP), and was independently correlated with histological changes which are typical of NAFLD, such as ballooning degeneration, lobular inflammation, steatosis and fibrosis degree (Yilmaz et al., 2015). Another study concluded that both NLR and mean platelet volume (MPV) differentiated subjects with NASH from those without this condition and that these non-invasive parameters presented compellingly higher values in patients with advanced liver fibrosis (F3-F4) when compared with counterparts with early liver fibrosis (F1-F2) (Abdel-Razik et al., 2016). Unsurprisingly, one meta-analysis of studies asserting a relationship between NLR and NAFLD suggested the integration of NLR into clinical settings for predicting significant liver fibrosis and NASH in individuals with NAFLD (Shavakhi et al., 2022). Still, there are also studies which failed to identify discrepancies in NLR values between subjects with hepatic steatosis and healthy counterparts, but which showed an inverse correlation between neutrophil-percentage-to-albumin ratio (NPAR) and liver attenuation values (Cucoranu et al., 2023). As a matter of fact, NPAR might perform better than NLR in predicting advanced liver fibrosis, in light of the results of a nationwide US study enrolling non-diabetic adults (Liu and Chien, 2023). Moreover, a Turkish study performed on adults proved that NLR does not correlate with severity of fibrosis and of hepatic inflammation, quantified through liver biopsy assessment (Kara et al., 2015).

NLR has been listed as a non-invasive biomarker which can predict mortality as well, in patients who require liver transplantation. One study including subjects with NAFLD showed that patients with NLR of at least 5 presented the highest mortality rate, within 3 months after being listed for liver transplantion (Leithead et al., 2015). Moreover, Kalra et al. proved that high NLR can determine the risk for cirrhotic decompensation, independently of Model for End-Stage Liver Disease (MELD) score and stage of cirrhosis (Kalra et al., 2017). In similar fashion, Maccali et al. concluded that the early identification of NLR increase, in the first 48 h of hospitalization, predicts short-term mortality for patients with acute decompensation of liver cirrhosiscaused by various etiologies, including NAFLD (Maccali et al., 2021).

## 5 The potential biomarker role of neutrophil-to-lymphocyte ratio in pediatric non-alcoholic fatty liver disease

Investigation of the role of NLR in predicting pediatric obesity, metabolic syndrome and their related complications gained more attention recently. Metabolic syndrome in children leads to a chronic inflammatory status, which is reflected by neutrophil increase and NLR elevation, according to Nicoară et al. (2023). NLR modifications in children with obesity seem to be reversible with appropriate intervention. One study proved that in those children adhering to a treatment plan consisting of behavioral changes, nutritional education and physical exercise, decrease in their standardized body mass index also led to mean decline in NLR values. This decrease has been also associated with improvement in ALT levels (Valle-Martos et al., 2023). On the other hand, Mansell et al. suggest that the persistence of adiposity-related chronic inflammation over time will not lead to significant changes in NLR values (Mansell et al., 2023). Moreover, one pediatric case-control study only reported a relevant increase in white blood cell counts in the obese study group, whereas neutrophil counts and NLR were similar between children with obesity and normoponderal counterparts (Mărginean et al., 2019). NLR also correlated with hyperlipidemia in children with type 1 diabetes mellitus (Salah et al., 2021). Other studies sustain that, unlike in adults, in children and adolescents NLR is not associated with metabolic syndrome, nor with its severity (Marra et al., 2023).

Duan et al. analyzed the ability of several inflammatory markers to distinguish children with obesity from the ones who had also developed NAFLD as a result of obesity. Within their study, no significant differences in NLR values were found between the two study groups, which also failed to correlate with NAFLD diagnosis, established upon sonographic features (Duan et al., 2022). In similar fashion, Kertmen et al. reported no differences in NLR values between obese children with steatohepatitis and those without evidence of liver fattening (Kertmen et al., 2018). However, neutrophil percentage and white blood cell count seem to be compellingly higher in obese children when compared to normoponderal counterparts, according to the study of Shi et al. Moreover, when a separate subgroup analysis was performed, on subpopulations with obesity and ultrasonographic changes of NAFLD, the difference in neutrophil percentage was significant only for children with simple fatty liver disease and steatohepatitis, whereas those without NAFLD presented similar NLR values to healthy controls (Shi et al., 2017). In the onset of liver dysfunction associated with childhood steatohepatitis, NLR changes have been associated with variations in ALT levels, but not with AST increase. However, these changes were reversible after 9 months of standard treatment and consequent ALT normalization (Valle-Martos et al., 2023). Still, pediatric data assessing the predictive role of NLR in pediatric NAFLD is scarce, as highlighted through Table 1, which represents a synopsis of available studies on this subject.

**TABLE 1:** Summary of studies which assessed NLR variation in relation to pediatric NAFLD.

(Reference author, year)	Type of study	Study population division	NLR outcome
Duan et al. (2022)	Case-control study	267 children	NLR did not differ significantly between the two study groups
		•176 NAFLD patients	NLR values did not associate with a NAFLD diagnosis
		•91 obese controls	
Kertmen et al. (2018)	Case-control study	104 children with obesity	NLR did not differ significantly between the two study groups
		•64 children with hepatosteatosis	
		•40 children without hepatosteatosis	
Shi et al. (2017)	Cross-sectional study	117 children with obesity	Children with simple fatty liver and steatohepatitis experienced significant increase in neutrophil percentage and statistically important decrease in lymphocyte percentage when compared to controls
		•23 children without NAFLD	
		•43 children with simple fatty liver	Neither neutrophil nor lymphocyte percentages did not differentiate children with NAFLD from children with excess weight and no evidence of liver impairment
		•51 children with steatohepatitis	
		209 healthy controls	
Valle-Martos et al. (2023)	Longitudinal study	63 children with obesity	NLR constituted an independent predictive factor of ALT changes
		•31 children with obesity who decreased their BMI after 9 months of standard treatment	Weight loss led to significant drop in NLR values
		•32 children with obesity who maintained a stable BMI	

## 6 Conclusion

Neutrophils are important effectors in the pathogenesis of NAFLD, bridging the inflammation which accompanies this condition, and constituting a particular abundant infiltrate within the portal region in children with NAFLD. The measurement of WBC subtypes and the derived parameter, NLR, might constitute an easily available tool which could particularly predict steatohepatitis development in children with obesity and could stratify the risk towards hepatic fibrosis progression in pediatric NASH. Further research is required to validate the biomarker role of NLR in pediatric NAFLD.
